# Animal Study Registries: Results from a Stakeholder Analysis on Potential Strengths, Weaknesses, Facilitators, and Barriers

**DOI:** 10.1371/journal.pbio.2000391

**Published:** 2016-11-10

**Authors:** Susanne Wieschowski, Diego S. Silva, Daniel Strech

**Affiliations:** 1 Institute for Ethics, History, and Philosophy of Medicine, Hannover Medical School, Hannover, Germany; 2 Faculty of Health Sciences, Simon Fraser University, Burnaby, Canada; Université Paris Descartes, France

## Abstract

Publication bias in animal research, its extent, its predictors, and its potential countermeasures are increasingly discussed. Recent reports and conferences highlight the potential strengths of animal study registries (ASRs) in this regard. Others have warned that prospective registration of animal studies could diminish creativity, add administrative burdens, and complicate intellectual property issues in translational research. A literature review and 21 international key-informant interviews were conducted and thematically analyzed to develop a comprehensive matrix of main- and subcategories for potential ASR-related strengths, weaknesses, facilitators, and barriers (SWFBs). We identified 130 potential SWFBs. All stakeholder groups agreed that ASRs could in various ways improve the quality and refinement of animal studies while allowing their number to be reduced, as well as supporting meta-research on animal studies. However, all stakeholder groups also highlighted the potential for theft of ideas, higher administrative burdens, and reduced creativity and serendipity in animal studies. Much more detailed reasoning was captured in the interviews than is currently found in the literature, providing a comprehensive account of the issues and arguments around ASRs. All stakeholder groups highlighted compelling potential strengths of ASRs. Although substantial weaknesses and implementation barriers were highlighted as well, different governance measures might help to minimize or even eliminate their impact. Such measures might include confidentiality time frames for accessing prospectively registered protocols, harmonized reporting requirements across ASRs, ethics reviews, lab notebooks, and journal submissions. The comprehensive information gathered in this study could help to guide a more evidence-based debate and to design pilot tests for ASRs.

## Author Summary

The manifold contributions over the last years on “publication bias” and “reproducibility crisis” in animal research initiated a debate on whether and how prospective animal study registries (ASRs) should be established in analogy to clinical trial registries. All recent debate, however, followed rather broad lines of argumentation and concluded that future decision-making on the issue of ASRs depends strongly on better knowledge about relevant characteristics of ASRs and about conflicting stakeholder interests. More qualitative but systematically developed evidence in this regard is needed. The primary objective of this study, therefore, was to present a systematically derived spectrum of all relevant strengths, weaknesses, facilitators and barriers (SWFBs) for ASRs. A systematic literature review and 21 key-informant interviews with experts from preclinical and clinical research, industry, and regulatory bodies were conducted to fulfill this objective. Our investigations resulted in a comprehensive and structured account of 130 issues and arguments around ASRs. Future debate and decision-making on ASRs might be heavily influenced by arguments and reasoning from individual experts and thus result in “eminence-based” policy making that relies on expert opinion. This study’s comprehensive spectrum of arguments and issues around ASR, developed through systematic and transparent methods, helps to balance the ongoing debate and thus facilitate a more evidence-based policy making.

## Introduction

In recent years, several reports have questioned the way animal research is conducted and reported, citing a lack of reproducibility of preclinical animal research data and poor translation of published preclinical data into the human setting [1,2]. Because results from preclinical animal research inform other preclinical research, early clinical research, and, in cases in which evidence from clinical trials is missing, even off-label clinical practice, accurate and complete reporting of animal research is essential to reduce harm to trial participants and patients, to optimize funding allocation, and to effectively reduce and refine animal research.

Incomplete reporting of studies and study results has been described as “publication bias,” “selective reporting,” and “dissemination bias” [3]. Whereas this bias has been studied in depth for clinical trials, less data is available for preclinical animal research. Various data sources have been used for the analysis of the publication rate of clinical trials: study protocols approved by an ethics committee [4–6] and study protocols registered in a trial registry [7]. The latter has become a much more useful data source since 2004, when the International Committee of Medical Journal Editors (ICMJE) made registration of clinical studies a prerequisite for publication in their journals [8]. In 2007 the United States Food and Drug Administration (FDA) Amendment Act further supported trial registration. The comparison of trial registries and journal publications has allowed new kinds of analysis: following the rate and time of journal publication, analyzing possible factors influencing publication status (such as study type and funding source), as well as finding discrepancies between registry entries and data published in journals [9,10]. This approach has helped to quantify dissemination bias in clinical research and to define possible reasons and solutions [11,12]. Similar studies are not available for animal research, because there are no registries for this type of study. Instead, analyses of dissemination bias in this field are more indirectly based on (A) data from survey research with animal researchers [13], (B) inferences from the debate about the relatively low reproducibility of preclinical research [14], (C) the high failure rate of early human trials [1,15], (D) statistical methods calculating the probability of bias in the available data [16,17], and (E) study abstracts published in conference proceedings or on their websites [18].

By analogy with clinical research, the implementation of prospective animal study registries (ASRs) has recently been suggested as one measure that might help to directly assess and substantially reduce dissemination bias in animal research [19]. A workshop on “publication bias in animal research” organized by the National Centre for the Replacement, Refinement & Reduction of Animals in Research (NC3R) in 2015 also focused on the issue of ASRs. A panel debate in this workshop demonstrated the broad lines of, and strong contrasts in, argumentation for and against ASRs. All panel participants agreed, however, that future decision-making on the issue of ASRs depends strongly on context, such as registry characteristics and knowledge about conflicting stakeholder interests.

This study aimed to address that need. The primary objective of this stakeholder analysis was to systematically and transparently assess the full spectrum of potential ASR-related strengths, weaknesses, facilitators, and barriers (SWFBs).

## Results

We present here an overview of the results of our stakeholder analysis; further interpretation of key results is then given in the Discussion section. Altogether, we identified 518 relevant text passages in the 21 interview transcripts (based on 13 hours of interview with stakeholders from four different groups, see the Material and Methods section) and 11 references [13, 19–28] (see also S2 Table), from which we derived 130 subcodes grouped under the four broad categories for ASR-related SWFBs. Table 1 presents our definitions for ASR-related SWFBs. The Material and Methods section further explains how the 130 subcodes were derived.

**Table 1:** Definitions for SWFBs of ASRs.

**Strengths**	The properties that enable ASRs to reach their intended goals. For our purposes, it also refers to the ethical arguments in favor of adopting ASRs.
**Weaknesses**	The properties that prevent ASRs reaching their intended goals or that lead to unintended effects. For our purposes, it also refers to the ethical arguments against adopting ASRs.
**Facilitators**	The circumstances that help ASRs to reach their intended goals. For our purposes, it refers to external or procedural factors that support the effective implementation of ASRs. “Effective” here means that the intended goals outweigh the potential negative effects.
**Barriers**	The circumstances that counter ASRs’ ability to reach their intended goals or that promote unintended effects. For our purposes, it refers to external or procedural factors that inhibit the effective implementation of ASRs.

**Fig 1:**
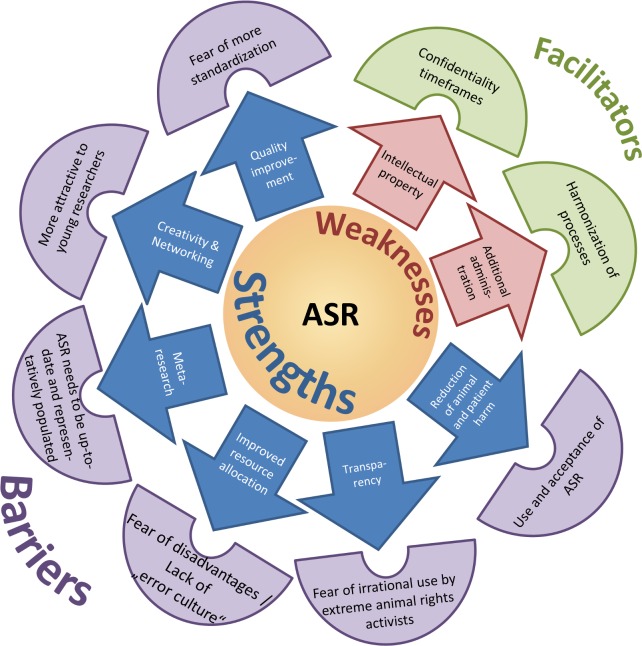
Visualization of core SWFBs.

Fig 1 visualizes core results and S1 Table presents the full spectrum of all 130 codes for the four SWFB categories together with sample quotations for each code (Fig 1, S1 Table).

For example, for the broad category “strengths,” our thematic analysis provided the subcode “Quality improvement, research support” by referring to the following two quotations: (A) “At the moment, I can publish a study with ten animals per cohort and you don’t know that I just ignored the other ten animals in the same cohort because I didn’t like what happened. There’s no way you could know that,” and (B) “In animal research, it’s really unusual for people to articulate a primary hypothesis. If you require study registration and the registries require that you stipulate a primary hypothesis, you are now creating a very strong motivation for people to actually start designing studies having thought through what their primary hypothesis is going to be.” S1 Table gives sample quotations for each of the 130 codes.

The thematic analysis reached saturation for the first level of subcategories, meaning that analyzing further interviews would not reveal other SWFB subcategories. For each SWFB category, we list subcategories separately by interest group: (a) animals, (b) preclinical/clinical research, (c) industry, (d) regulators, (e) public/patients, and (f) overarching issues. Most codes were related to “preclinical/clinical research” (*n* = 63), followed by “overarching issues” (*n* = 25), “industry” (*n* = 15), “public/patients” (*n* = 10), “animals” (*n* = 7), and “regulators” (*n* = 4).

## Discussion

This study presents the full spectrum of 130 potential SWFBs for ASRs based on 11 publications and 21 key-informant interviews with experts from all relevant stakeholder groups. These systematically developed qualitative findings might serve as a comprehensive source for future discussion, pilot-testing, and decision-making on ASRs by researchers, funding organizations, scientific journals, regulatory bodies, and the public.

Our qualitative findings do not allow quantitative, generalizable conclusions such as “90% of researchers but only 40% of regulators agreed that…” Quantitative survey research would be needed to draw such conclusions. At this stage of a relatively young debate, however, we believe that knowledge about the full spectrum of potential SWFBs should be of core interest to all stakeholders. Furthermore, the development of future survey questionnaires on ASRs should ideally be based on such knowledge. Last but not least, those who express their attitudes towards ASRs in current debate and in future opinion polls might give different answers if they are first informed about all potential SWFBs as presented in this paper.

Having said this, we will in the following discuss which of the mentioned issues were “relatively” controversial (on which opinions and arguments diverged among interviewees) and which appeared to be “relatively” uncontroversial (on which many interviewees explicitly or implicitly agreed). However, the reader should bear in mind that, as we are reporting findings from qualitative research, no inferences can be drawn about the prevalence of controversies or consensus observed beyond the sample.

### Uncontroversial Potential Strengths

Interviewees across all stakeholder groups said that a registry would most probably function as an incentive for (i) more rigorous study protocols for animal research (codes S9a and S9b in S1 Table), (ii) less selective reporting in journal publications (code S10), and (iii) less biased reporting of preclinical data in protocols and investigator brochures for early human studies that are submitted to Research Ethics Committees (RECs) or regulatory bodies (code S34).

It was also undisputed that a registry could help to better disseminate evidence (code S4a), thereby promoting transparency (codes S33 and S5) and also facilitating network building among researchers who work on similar research questions (code S6a). How strongly these potential strengths would materialize was difficult for the interviewees to anticipate, but it was highlighted that if a qualitatively appropriate and informative registry such as clinicaltrials.gov existed for preclinical research, this could serve as a core information source for searching and refining ideas (code S4b), fostering networks (code S6b), disseminating findings (codes S34), and promoting trust within the scientific community (codes S19a and S19b).

Interviewees also unanimously described as a strength the role of registries in facilitating meta-research (research on preclinical research) that could, for example, help to quantify publication bias in a more robust way (codes S14 and S15a).

### Controversial Potential Strengths

Based on experience of clinical trial registries, all stakeholder groups were ambivalent about whether ASRs would directly reduce publication bias (codes S10 and S15b) or whether it would rather be a tool for other more indirect methods, as described above.

Sounder research funding allocation (code S32) was also highlighted as a potential strength. This is because reviewing research grants can also be affected by biased publication; decreased bias could thus improve judgments on whether a research proposal really suggests new and relevant investigations. Similarly, some interviewees pointed to a positive impact on replacement, reduction, and refinement (3R principles) of animal experiments (codes S2a, S2b, and S3). However, others feared that stricter regulations following an ASR implementation, e.g., requirements to meet sample size calculations, might in the end lead to larger study group sizes and therefore to more animals being used in preclinical animal research (code B4). On the other hand, this could increase the statistical power of animal studies performed (code S9a). The overall numbers of animals as well as the cost might be balanced by the fact that fewer redundant experiments would be performed (code W2). With regard to this concern, other interviewees highlighted the need to better differentiate between exploratory and confirmatory animal studies [29]. Whereas the registration of confirmatory animal studies would help reduce waste in clinical research (codes S26a and S26b), the registration of exploratory studies would rather help avoid the redundant use of animals (codes S2b and S2c) and provide information that might help in the refinement of animal studies (code S3).

Another controversial topic was centered on whether and how ASRs might affect financial, human, and time resources (code S18a). We describe potential strengths in this paragraph and related weaknesses in the next section. Some interviewees suggested as a potential strength the savings in time and money if ASRs helped avoid experiments already performed elsewhere (codes S2b and S18b). Some industry representatives argued that this might also reduce the cost of drug development (code S25), whereas others were skeptical due to the complex reasons for high costs in competitive drug development (code W10).

It was also controversial whether public trust in biomedical research more generally and in animal research specifically could be increased by transparency resulting from ASRs (codes B1a and B2a). It was mentioned that the public will always welcome more transparency (code S28b), but interviewees remained skeptical about whether ASR-related transparency will finally result in more or less public trust in research (codes S28a and B1b). See the “Barriers” section for more information on this issue.

### Uncontroversial Potential Weaknesses

A clear weakness for researchers in both industry and academia was the negative impact on intellectual property (code W3) and the associated potential theft of ideas (code W4). See the section “Uncontroversial Potential Facilitators” below for potential solutions offered by interviewees to this issue. Addressing this concern requires balancing intellectual property interests that demand a confidentiality time frame as long as possible with the usefulness of a registry that requires detailed and up-to-date information.

Another fear expressed by animal researchers was of the additional administration and the time needed to accomplish it (see codes W1a and W1b). Some mentioned the considerable amount of time they already have to invest in complying with regulation and documentation requirements. However, participants assumed any registry would be time consuming, and they recognized that there may be ways to reduce expenditure of time, as explained in the section “Uncontroversial Potential Facilitators” below. One interviewee highlighted the difference between the additional time needed for administration and the time saved by reducing unnecessary repetitions of experiments (code S18c), which might explain the initially surprising emergence of time issues in both the strengths and the weaknesses category.

### Controversial Potential Weaknesses

Some interviewees argued that ASRs will negatively affect creativity and serendipitous findings in animal research (code W6), fearing that a registry could preemptively define a structure for studies that might be too rigid to capture some project ideas and thereby prevent certain types of research (code W7). However, those who agreed that a registry could negatively affect creativity mainly referred to exploratory research, which they did not want to be limited by a time-consuming registry. Others also highlighted how more comprehensive and less biased information on previous research might even facilitate creativity and inspire innovative research questions (code S7).

### Uncontroversial Potential Facilitators

The possible theft of ideas and the associated competitive disadvantage was an issue that worried both academic and industrial researchers. However, when it was suggested that registry entries could be set for disclosure at some future moment to protect intellectual property, many interviewees agreed that, depending on the time frame (for which one to two years was often suggested), this would facilitate an ASR implementation (codes F9a and F9b).

Although the administrative burden was a frequently mentioned weakness, one possible solution to this was mentioned as well: if the “core data” for an ASR was congruent with the data that need to be submitted for research funding, approval processes, journal submissions, and other purposes (e.g., using the ARRIVE guidelines as a basis), the time constraints would be lessened greatly (code F40). In addition, some interviewees highlighted that digital lab notebooks (DLNs) could play a central role in quick and efficient registration of studies: “If you have all your study characteristics on your DLN, then you just need to press the submit button to upload your protocol to a registry” (code F11b). However, interviewees were rather pessimistic as to whether such harmonization of core data and the implementation of DLNs are realizable in the next few years. The importance of the resource question is illustrated by the fact that several facilitators mentioned by animal researchers were related to financial or staff support to cope with the additional workload (codes F12 and F13).

### Controversial Potential Facilitators

Again, regarding the topic of intellectual property, a few interviewees suggested retrospective registration as a possible solution (code F8). However, the use of such a retrospective registry was questioned when it came to possible strengths in quality improvement, such as reduction of biased data or incentives for better study design (codes S8–13).

A strong debate arose around the question of voluntariness. Interviewees did not agree whether voluntary registration would yield a database contributed to and used by many people (code F27) or whether enforcement, e.g., by publishers or legislation, was needed to create a well-populated registry useful to the research community as a whole (code F19). The example of clinical study registration, in which after the publication of the ICMJE statement on registration as a prerequisite for journal publication an enormous increase in clinical trial registration was observed [30], suggests that some kind of incentive or enforcement is needed to push forward the implementation of such a tool.

### Uncontroversial Potential Barriers

A point mentioned by some of the animal researchers was the fear of disadvantages in funding or career development, especially for scientists appearing with many “negative” results or failed studies in such a registry (codes 15a and 15b). This often led to comments on the lack of a proper “culture of error” in preclinical research (codes 14a and 14b). Many interviewees affirmed this comment and highlighted in this context that registries could help to shed more light on the obvious issue that research only improves via failures [31]. The feared transparency of failure could also influence the creativity issue, because researchers might prioritize “safe/low-risk” research questions rather than “innovative/high-risk” questions in order to avoid “negative” registry entries.

### Controversial Potential Barriers

The question of time and personnel resources also emerged as a possible barrier due to the lack of time needed to effectively use a registry both by researchers (codes B6 and B30) or by RECs (code B28). However, at least among the researchers, many interviewees emphasized the possible time savings from avoidance of studies that had already been performed or experimental set-ups that have proven unsuccessful (see “Uncontroversial Potential Strengths”).

Some researchers feared the irrational use of the registry by animal rights activists (B1c) and therefore would prefer a registry that either doesn’t show names of the scientists or that is generally not open to the public (codes F25 and F26). Other interviewees countered these proposals with the arguments that names of animal researchers and the experiments they have performed are increasingly publicly available, e.g., through open access publications, and that transparency and proactive information of the public may increase public trust (codes B2b and B2c).

As a more general barrier, some of the potential strengths of a registry, such as networking possibilities, better visibility, and improved resource allocation, seem to be more attractive to young researchers rather than established group leaders for whom the benefits might be outweighed by disadvantages, such as the possible loss of competitive edge and the fear of more standardization, regulation, and administration (codes S19c, F22, and B18). A similar effect was mentioned in the industrial realm, in which the big, established companies are more likely to see competitive disadvantages in a registry, whereas for smaller companies, the benefits might prevail (code F33).

In summary, our interview study showed that there is broad interest among all stakeholders in increased transparency in preclinical animal research. ASRs might play a crucial role in this regard. As usual, the devil is in the detail, and it depends on the registry structure and on implementation and framing conditions how well this tool would balance potential strengths and weaknesses and how it would be accepted in the scientific community. Furthermore, the question arises whether there are subgroups of animal studies that are more or less suited for registration, or that benefit the different stakeholder groups distinctly.

Although some kind of regulation may be needed to put this into practice, it is also important to protect the interests of the affected stakeholder groups, maybe by setting a confidentiality time frame in which prospectively registered information is not accessible to others and competitive advantages are not compromised.

Whether more transparency via ASRs could speed up the process of drug development is hard to predict. This, of course, would be in line with the interests of all stakeholders and would add to current developments in the pharma industry to stop the expensive development of ineffective pharmaceuticals as early as possible. Finally, there are already efforts from the pharma industry similar to the idea of ASRs, albeit only for certain fields, such as toxicology (e.g., Registry of Industrial Toxicology Animal-data [RITA]; see http://reni.item.fraunhofer.de/reni/public/rita/).

As a next step, pilot registries could be tested to assess the kind of information and the level of detail needed in an effective and efficient ASR. As one interviewee said about ASRs, “One cannot kill good ideas, and the idea of transparency is a great one.” Improved transparency is currently being discussed in several research domains [29,30], but of course the research community needs time to become familiar with the associated concepts. Therefore, the stakeholders involved in animal research and affected by an ASR implementation should take the chance to participate in the discussion and to shape the future of their field.

## Material and Methods

### Ethics Statement

The Hannover Medical School REC approved the study, and all interview participants provided written informed consent.

### Literature Review

An exploratory search of relevant literature was made using PubMed in June 2015 with two search strings, “preclinical stud* regist*” and “animal stud* regist*,” resulting in 175 and 388 hits, respectively. All titles were screened to identify papers addressing the issue of ASRs or closely related issues. Further papers were obtained via consultation with experts in the field. Snowball strategies (reference check and citation check) for the included papers were applied using Scopus and Google Scholar but did not reveal other relevant literature. Because of the small number of finally included references (*n* = 11, see S2 Table), of which the majority did not provide detailed information but merely stated that an ASR was a potentially important means of addressing the problem of publication bias in preclinical studies, we conducted key-informant interviews and used them as our main data source for the stakeholder analysis.

### Key-Informant Interviews

We performed semi-structured, open-ended interviews with “key informants,” that is, persons being an “expert source of information” [32]. The following five criteria helped to define key informants: role in the community, knowledge, willingness, communicability, and impartiality [32]. We invited experts from different stakeholder groups that proved via authorship in relevant publications that they are “knowledgeable.” From workshops on the topics, such as the NC3R workshop in 2015, we were also able to identify potential interview participants that are “communicable” and “willing” to express their viewpoints on the topics.

Our key informants belonged to four different stakeholder groups, intended to represent the groups most affected by or most influential on the implementation of an ASR, and were selected by purposive sampling. Our sampling was purposive, as we aimed to address the diversity of existing viewpoints and relevant expertise, and we aimed to interview “information-rich” stakeholders. In our case, we wanted to recruit key actors from industry, targeting product developers/researchers, as well as people from overarching organizations. For researchers, it was important to include different research areas as well as career stages.

1. AR = Animal Researcher (e.g., postdoc, animal welfare officer, head of animal research facility, *n* = 9)
2. CR = Clinical Researcher (e.g., deputy from a clinical research unit with 40 beds for phase I/II research, head of a clinical research facility, *n* = 2)
3. I = Industry (e.g., chief/top executives from an industrial association and from pharmaceutical companies, *n* = 4)
4. RE = Regulation and Ethics (e.g., chief/top executives from federal agencies and from a study registry, researcher in biomedical ethics, *n* = 6)

The interview guide (S1 Text) started with open questions on potential strengths and weaknesses of ASRs from the viewpoint of the respective stakeholder group. Over the course of the interview, further questions regarding potential facilitators of and barriers to ASR implementation were added. The interview guide was discussed with external experts and pilot-tested for feasibility and acceptability in two cognitive interviews. All interviewees were sent the same invitation and signed a written consent form (S2 Text and S3 Text).

Of the 21 interviewees, 14 were from Germany, 3 from Great Britain, 2 from the United States, 1 from Singapore, and 1 from Canada. Interviews were performed in person or via telephone and lasted 40–45 minutes. 13 interviews were conducted by DS, 5 by SW, and 3 by DSS. All interviews were audiotaped and transcribed.

### Coding Process

To extract, analyze, and synthesize the relevant information on SWFBs for ASRs, thematic text analysis was applied to all 21 transcripts and 11 references using MaxQDA [33]. See Table 1 for our definitions of SWFB. Having conducted 15 interviews, first, 3 transcripts were systematically analyzed by all authors independently. Interview passages mentioning SWFBs were identified, and a descriptive code was applied. Second, the findings were compared to identify potential differences in coding. However, only minor differences occurred and were solved by discussion. Third, aspects mentioned in one interview were matched with those from another in order to collate the various codes and to cluster the findings into an initial matrix of categories and subcategories for SWFBs. This matrix served as a starting point for the further thematic analysis of the other 12 transcripts and the 11 references. One researcher (SW) employed the above-described approach to add and modify codes until preliminary thematic saturation was achieved for the main categories and first-order subcategories. Thematic saturation implies that no new categories (themes) can be generated for the SWFB matrix, which is itself the primary result of the thematic analysis [34]. This resulted in a matrix of broad and narrow categories for SWFBs. Another researcher (DS) then checked all transcripts and references and the resulting matrix and proposed changes. After agreement on the preliminary SWFB matrix, we conducted six more interviews to verify that we had reached thematic saturation. The analysis of the six additional interviews added information that could be grouped under the already existing categories, and we also slightly modified the wording of existing categories. However, the second round of interviews did not result in additional categories or major modifications to the SWFB matrix. We thus confirmed thematic saturation. All researchers discussed and slightly modified the matrix for internal consistency and agreed the final matrix.

## Supporting Information

S1 Table Sample quotations. (DOCX)

S2 Table Literature review. (DOCX)

S1 Text Interview guide. (DOCX)

S2 Text Invitation letter. (DOCX)

S3 Text Consent form. (DOCX)

## Abbreviations

ASRs: animal study registries
DLNs: digital lab notebooks
FDA: United States Food and Drug Administration
ICMJE: International Committee of Medical Journal Editors
NC3R: National Centre for the Replacement, Refinement & Reduction of Animals in Research
REC: Research Ethics Committee
RITA: Registry of Industrial Toxicology Animal-data
SWFBs: strengths, weaknesses, facilitators, and barriers
