# Supplementary material for: Animal Study Registries: Results from a Stakeholder Analysis on Potential Strengths, Weaknesses, Facilitators, and Barriers
Source: PLoS Biol. 2016 Nov 10;14(11):e2000391. doi: 10.1371/journal.pbio.2000391 (PMC5104355; doi:10.1371/journal.pbio.2000391)
Supplement: S1 Table — (DOCX) [file pbio.2000391.s001.docx]

**A****nimal study registries. Results from key informant interviews on potential strengths, weaknesses, facilitators and barriers**

Susanne Wieschowski^1^,PhD, Diego S. Silva^1,2^, PhD, Daniel Strech^1^, M.D., PhD

1 Institute for Ethics, History and Philosophy of Medicine, Hannover Medical School, Carl-Neuberg-Strasse 1, 30625 Hannover, Germany

2 Faculty of Health Sciences, Simon Fraser University, Blusson Hall, Room 11008, 8888 University Drive, Burnaby, B.C., Canada, V5A 1S6

Corresponding author:

Daniel Strech, Institute for Ethics, History and Philosophy of Medicine, Hannover Medical School, Carl-Neuberg-Strasse 1, 30625 Hannover, Germany

Phone: +49 511 532-2709/-6498

E-Mail: strech.daniel@mh-hannover.de

Table S1: Sample quotations

| **Strengths** | | | | | | | |
| --- | --- | --- | --- | --- | --- | --- | --- |
| **Animals** | General issues | | | | S1 | S1 | AR4 Well I am a veterinary doctor and I am more "pro animal". However I can imagine that other scientists see things differently. But for me, I'm thinking for the animal and for animal protection and therefore to me it is a matter of course to contribute something to this. |
|  | Reduction | | | | S2 | S2a | RE1 I didn’t know from the others of course and I don’t know how many mice had to die without this really being necessary. Because trials were redone and the knowledge that things didn’t worked had already been in some people’s desk |
|  |  |  |  |  |  | S2b | AR 4 This is obvious. I think you can greatly reduce animal experiments and save costs, also for yourself. Because an animal costs money, as well as the experiments. And if I know someone has done this before, I can just save this. |
|  |  |  |  |  |  | S2c | AR2 In literature you found papers saying, it works like this, do this, do this, do this and it sounds really easy. And sometimes we tried it and we were not successful. And then we invited some other group that is working on this issue of reproducibility in [species] and they came and try to show it and then somebody said, “Yes it doesn’t work with us either”. And we said, “But it was written here”, “Yes that was one or two, this was…”. And that is something really interesting, that they have struggled at the same point like us. But you cannot see that in the literature. We could have just forget all of our trials before if we have talked to them before or had this information before. |
|  | Refinement | | | | S3 | S3 | AR2 for example if you’re planning in infectious study, so you infect a mouse with a specific germ that is pathogen to human, so you need to find a therapy, so you have infect the mice, you have no idea what doses concentrations of antibiotic do I have to take. So you start with a very high and a very low, and somewhere in between you will find the exact concentrations that will heal the animal. So I need to know for example, the highest doses are toxic for the animal, not only for the germs, but also for the animal. So it will be really good to know that before, when somebody else already tried it. So I can say that “Okay, that concentrations of the antibiotic or other things is toxic to my mouse so I don’t have to try it again”. |
| **Preclinical/ Clinical research** | Production and dissemination of evidence | General issues | | | S4 | S4a | CR2 That means there are often bad data or no data at all and it would help enormously if the head of a clinical study program could determine in a fast and valid way what data there is worldwide, what has been done already and what not, since mostly there are data lying in the drawers of companies and no one has access. |
|  |  |  |  |  |  | S4b | I3 If one could find out easily what kind of experiments that I have in mind have been done there or I have read publication X and am looking for similar or identical data now, then I would get valuable information out of it. It could, e.g. contextualize a publication, add more details or confirm that there is something to the key message of the publication. Thereby I would have more evidence before investing in a new topic. |
|  |  | Transparency | | | S5 | S5 | I2 This transparency puts everyone under pressure to work correctly |
|  |  | ASR as an exchange platform | | | S6 | S6a | AR4 On the other hand, maybe someone sees this and talks to the researcher, maybe they work on a similar topic and they could talk about it; they could say, we could sit together and work it out. |
|  |  |  |  |  |  | S6b | RE1 the researchers themselves, the young scientist they could just show the work what they are doing, even if they don’t get published in the big journals but they have international database like again WHO or any other institution affiliated so that everybody sees it and everybody can read it. They can have contacts and they can get ideas from the others |
|  |  | Improvement of creativity, new ideas | | | S7 | S7 | AR2 It could impact creativity? No, I don’t think so actually. Because if you’re a researcher and you’re in a special issue then you have your own ideas. Maybe it would, on the contrary, increase the creativity. Because you’ll get maybe a new ideas or other points to look for. I would say the other way round. It’s not impeding creativity. |
|  | Quality improvement, research support | General issues | | | S8 | S8 | I4 So, with that in mind, I think anything that helps moving forward would be useful and helpful and I think at the highest level in general terms an animal registry would be helpful. We do need to understand the specifics of what that would means but I think that anything that tries to improve the focus on how an experiment is performed rather than the result that comes out of the other end would be helpful. |
|  |  | Incentive for better study design | | | S9 | S9a | AR7 So, I think there would be a shift in the awareness of quality of how we draw experiments and the impact of quality on results. And we’d see definitely a change in the reported quality anyway of experiments, which I think I would guess means that there is a change in the actual quality of the experiment. And I think part of that is the amount of evidence that shows you that things are important and I think if you had to preregister, it fits in, because we are trying to address the reproducibility issue ---- and I think it fits into that, a kind of umbrella of how can we make… If you’re the kind of person that had to preregister your animal study, then you’d think about reproducibility and how can we make experiments more reproducible. You don’t want to be the one person who doesn’t have an experiment that is randomized and blinded or is underpowered. |
|  |  |  |  |  |  | S9b | RE3 In animal research it’s really unusual for people to articulate a primary hypothesis. If you require study registration and the registries require that you stipulate a primary hypothesis, you are now creating a very strong motivation for people to actually start designing studies having thought through what their primary hypothesis is going to be. |
|  |  | Reduction of biased data | | | S10 | S10 | I4 At the moment I can publish a study with 10 animals per cohort and you don’t know that I just ignored the other 10 animals in the same cohort because I didn’t like what happened. There’s no way you could know that. |
|  |  | Minimization of P-fishing; P-hacking | | | S11 | S11 | AR9 That is where it starts: people don't even have primary outcomes. They do 20 or sometimes they even have more endpoints than mice. And everything is tested [for significance] without correction for anything and in the end they just grab those that by chance were positive. All this would cease or wouldn't work any longer if this part had to be registered. |
|  |  | Interpretation of studies/data | | | S12 | S12 | RE3 So, one of the major sources of publication, of bias in research is that people make modifications to their protocol in a manner that brings out the positive aspects of the story and subordinates the negative aspects of whatever they have found. So, having greater transparency about the way a study was set up allows users of that information to know or at least to make inferences of whether or not a finding is probably a robust finding or whether or not the finding may be affected by some sorts of biases in a way that the design was adjusted as the study was pursued. |
|  |  | Further development of old substances | | | S13 | S13 | CR1 These are old substances one wants to reuse in a new indication. And for those substances there would be a huge benefit if one could see, 5 or 10 years ago someone did this and these were the results. I don't have to do it again or I am doing it again with some modifications or in a different model. Or it had an effect but there was no money for bringing it into clinical practice and I want to do this now. |
|  | Research on research | General issues | | | S14 | S14 | RE3 So the other big virtue of transparency is that it allows one to do research on research. So the literature on clinical trial registration is pretty thin with respect to how registration has actually improved research practices. So we don’t actually know that the advent of prospective of trials have actually meaningfully changed publication practices in research or whether it has meaningfully changed the ability of people to form valid interpretations of studies that are published. But we do know for sure that transparency via registration has allowed a cottage industry of research on research, what people are now calling meta research. And it’s really through those kinds of studies that we begin to understand of the mechanisms through which biases get expressed or propagated in research. |
|  |  | Quantification and characterization of pub bias | | | S15 | S15a | RE2 if you registered every study that was ever carried out, then that will give you the means to quantify publication bias to start with, because actually we have very limited data about publication bias in preclinical research, |
|  |  |  |  |  |  | S15b | RE2 I have never seen clinical trial preregistration presented as a real means to reduce the publication bias. But at the end of the day, we know that it helps quantify it but it doesn’t really reduce it. If you look at the clinical field, it is not… there is no clear evidence that it has reduced the publication bias and it’s not the real aim for it - the aim is actually trying to quantify the problems rather than reducing it. |
|  | Resource considerations | General issues | | | S16 | S16 | I3 Before investing in a certain area I would have more evidence. |
|  |  | Saving money | | | S17 | S17 | AR8 Looking at the economic damage, the publication of non-positive results is as important as - or even more important than - that of positive results. Simply the financial and human resources spent on studies that have been done before, reproducing the negative results - this is ridiculous. |
|  |  | Saving time | | | S18 | S18a | AR2 You can see what others have already done, where the failures are so you can conduct your experiments in a fast way to get results. |
|  |  |  |  |  |  | S18b | AR4 In my personal experience: when you start an experiment and you start looking into the literature for what has already been done and you find nothing then you ask yourself, has this been done before but didn't work or has just nobody done it before? Because you find no previous results you start your experiment and it doesn't work, the substance doesn't show an effect and you don't publish it yourself because you don't know where to submit it. |
|  |  |  |  |  |  | S18c | AR7 Yes, so I think that time is an issue, but then I think you could argue that if I’m not doing an experiment that someone else has done because I know it has been done because it is on a registry then that would save a lot more time and money than the cost of you know entering data into a form. But that is a much more kind of citizen science approach, because it’s probably not my individual time I’m saving but time on a grand scale. And not everyone thinks that way. So that might be a bit of utopian thinking about it. |
|  | Scientific professionalism; community trust | | | | S19 | S19a | RE3 One of them is that allows people that when they read studies to be confident that the researchers have pursued a study consistent with what they have planned. |
|  |  |  |  |  |  | S19b | AR2 So they’ll say “Okay this is something worth for other researchers to read, to know, then I will put it into the registry like that, for everybody to use”. {Interviewer interjecting ‘right’} I mean it’s not the way that everybody just wanting to shield the errors or mistakes and so on. So there are always projects or studies with unexpected results that you think “Okay, everybody else in this field should know about this but there’s no way to publish it because they can’t publish in the paper that something went completely wrong”. |
|  |  |  |  |  |  | S19c | AR4 Not that negative, not like this didn't work and that didn't work, but more like a sort of publication that counts as well. Because you did something, even if it didn't show the results you expected. Because also negative data can take you forward. I know it's a big discussion with the journals as well. And maybe the positive aspect, that you used your time to type your data into the registry. |
|  | Sort of marketing platform | | | | S20 | S20 | RE 1 all those little biotech companies they were very eager to register with us, never said ‘oh my god our IP, what should we do? And others will compete with us?” They have thought of the registry some sort of - and it was 2001, 2002, long before the registries were really implemented, or the regulation was implemented - and then thought of it more, sort of marketing platform for people know what we are doing. |
|  | Protecting intellectual property by registration date | | | | S21 | S21 | I4 And if I register my study before you register yours, it’s clear that my idea was first and you copied me. Simple. And that’s how patent law works. |
| **Industry** | Improved insight into scientific community/ASR as exchange platform | | | | S22 | S22 | I3 I believe that what I get here through full integration into the scientific community more than compensates for the seeming loss in competitive advantage. |
|  | Equally distributed access to potential drug targets | | | | S23 | S23 | RE1 And if you look at what is really new in medicinal product, you know the figures, there’s nothing really new, very little. So they are more or less dependent on their spies on the field, their contact of the academia, I think the registry like that could help. If you do it right. |
|  | Resource considerations | General issues | | | S24 | S24 | I2 Maybe the economic argument would even be that we could prevent people investing their time and intelligence into projects that are pointless anyway. |
|  |  | Investing money in well-characterized substances | | | S25 | S25 | I3 Just a guess: an idea inspired by a paper, until an internal validation is performed, to go into the next phase, the high-throughput-screening, costs 200,000-300,000 Euro. The high-throughput-screen then costs me 1 million euro, then the compound needs to be refined, let's say again 200,000 Euro on top, so 1.5 million Euro in the end. Now I test the substance in my animal model - and nothing happens. I stop the trial and 1.5 million Euros are gone, that would maybe not have been spent, if I had the whole picture including negative results beforehand, which have not been published. How often do we have this? Let's say it is half of the cases and I just take numbers now which are easy to round, so let's say 10 times a million Euro, so 10 million Euro are gone. So a medium-sized to big company loses 10 million Euro a year for findings they cannot reproduce in such an early phase already. If it was possible to get the full picture and to prevent only, I'm guessing again, half of those cases then you could save 5 million Euro. That is something. |
| **Patients/ Public** | Reducing risk of harm | General issues | | | S26 | S26a | AR1 So more thorough preclinical studies might give better result. You know, which can be translated to the patient. At the end of the day, we still cannot say, because animal are a different patient, they are different. |
|  |  |  |  |  |  | S26b | CR2 If for example Tegenero had been pharmacologically investigated in primates before, then one could have avoided those severe side effects in humans. That means, the 7 subjects in England that suffered from severe damage could have been avoided if there had been animal experiments like this before and from a registry they would have known enough. |
|  |  | Support for ethics review | | | S27 | S27 | RE1 We assume that those ethics committees look at those registries before they discuss about a submitted new clinical trial but that’s not the case. That would be of course very helpful, it was one of the reasons we did that, so in the same of course true with those studies, I mean if you do your work well and have time and resources for it, if you have to really look at an application, you should know what has been done already, and again not what’s the researcher said but which you can verify |
|  | Public Trust | | | | S28 | S28a | I2 Nevertheless I would appreciate if the public had the right, the opportunity to have a look at this. So, for example, someone suffering from a certain disease [...] who wants to know what progress the development of therapeutics has made that I might someday benefit from. And what is behind it? Of course it would only be very few people using it, but I don't see a counter argument unless you want to cheat. |
|  |  |  |  |  |  | S28b | RE2 I think the public would welcome a registry like that. Not so much in terms of I want to read it or I want to understand it but more in this feelings safe when things are transparent. |
|  | Resource considerations | General issues | | | S29 | S29 | AR1 And I think at the end of the clinical trial as well. Then it comes to the question, what is going wrong, why is it that our clinical trials are not successful enough. Because we have so many companies, so many new drugs that we have produced, so many things we’ve done, so many clinical trials, thousands of animals have been shown to work then somehow when it comes to the patients it doesn’t seems to work that well, so we can prevent a lot of wastage and trying to solve the problem at the preclinical level. Standardize the procedures so that the result we get then can be used successfully in clinical trials. |
|  |  | Faster drug development | | | S30 | S30 | I2 It probably means as well the development of other industries, like already today we are seeing specialists for phase 1 and 2 or preclinical research, who have their portfolio of products they sell, and the whole process of pharmaceutical development will be much more divided, which is starting already now. So there won't be one Pfizer, developing a product from the first idea, maybe collecting, let's say snake venom in Africa, to a marketable product 10 years later. Instead there will be companies doing only one step, e.g. selling molecule ideas or other developmental stages. Everyone will be much more under pressure to be fast, because the second they start everyone else will know as well, therefore you can't play around for five years but you have to bring it to the market instantly. So there will be an acceleration and a lessening of monopolies. And that isn't bad from the population perspective. |
|  |  | Increasing value of research funding | | | S31 | S31 | RE1 I think it would be rather important than not important, because, when I look at the what kind of resources and grants are applied for here that is really big dimension and half of it is not published |
|  |  | Improved allocation of research funding | | | S32 | S32 | RE1 for the… they who give the grant would important as well because they will never know what is been done what has already been done and in trials they now try to get the bigger picture and to know and to be up to date on what’s already been done but in those early trials there’s always of course dependent on what the researcher writes in his grant application: “this has never been done and I’m the first to do that, this will always work, it worked in cell cultures so it work in mice”, yes you say so, yes I think that would be very helpful those and for allocating the money to the right places |
|  | Information Source | | | | S33 | S33 | I2 But democracy means transparency and only a transparent system can be democratic and that means at least that each citizen has the possiblity to get this information. |
| **Regulators** | Improved knowledge on existing studies | | | | S34 | S34 | RE4 As a regulatory agency we would see a strength in knowing what studies have been performed or which are planned and we would have the advantage that concealment of negative studies or negative results is made difficult because we can ask then, what about study X, what were the results. |
| **Journals** | Validation of claimed methodologies | | | | S35 | S35 | I4 And when I’m submitting to Nature, I can check all of those things, the study was performed blinded, the animals were randomized. But I do that after the study. You don’t know that I really did that. There is no way you’d know I really did that. I just can check the box and there is no way of knowing. The only way for you to know is if my university checks my lab notebooks. Because Nature is not going to do that. So you know, the very least, we got to have the journals, the funding agencies, the institutions all cooperating. I think this is probably a useful step going forward. |
| **User groups that were not anticipated** | General issues | | | | S36 | S36 | RE1 registries are used by groups we haven’t thought off before like the insurance company. Say “dear patients, I can’t pay for your therapy but there is trial going on in such and such city where they use that, so please go there and you will have it for free”. Things you have never thought about will happen right? |
| **Weaknesses** | | | | | | | |
| **Preclinical/ Clinical research** | Resource considerations | Additional administration | | | W1 | W1a | AR5 We are researchers and we only want to do research. Over the years we have been burdened with more and more bureaucracy, for S1 labs, for radioactivity, for chemicals, and so on. Sometimes I can hardly leave my desk at all and before starting an experiment I have to consider so many things, whether I filled in this form or whether I registered there, it's just impossible in the time I have. |
|  |  |  |  |  |  | W1b | AR6 When I hear that there is again something additional, then I'm a bit concerned. Because we already have to fill in a lot of tables and overviews and whatever. In fact, we had to hire additional staff to cope with this. |
|  |  | Increasing costs by increased sample sizes | | | W2 |  | I4 I think that the biggest problem we have is that animal studies are too small. There are not enough animals used in the studies to generate a result which we can really believe. So that might actually increase the costs of an animal study, it will certainly decrease the number of studies, so might balance that. |
|  | Intellectual property | General issues | | | W3 | W3 | RE2 There might be further complications if you think about the confidentiality of the research. If you want to protect… if you looking into journal, you might want to patent later on… you want to protect your method or you don’t want your competitors to do the same thing. There might be an issue of complication in that respect. And maybe you wouldn’t want to give out too much detail. |
|  |  | Potential theft of ideas | | | W4 | W4 | RE1 Well in clinical trial I think most people now realized that it’s hard work to do from the scratch the clinical trial and then copy someone who is always a couple of years ahead of you, but in preclinical trials is of course far easier to test just one enzyme or one particular bio marker, and to just re-do with the same experiment and I can really understand the fear that you are copied and other people with more resources, more people are then over taking… and quicker than you. |
|  | Interferes with current career pathways | | | | W5 | W5 | AR5 It's extremely difficult, the competition is so strong. And as long as your success as a researcher depends on impact points, your financing depends on impact points, your future position as a professor or similar in a research institute depends on impact points, this is not going to change. |
|  | Loss of creativity | General issues | | | W6 | W6 | I3 I am totall pro registries for clinical trials, I think it is a must there, but it is an extremely regulated environment anyway, very process-oriented, where the studies, even the small ones, are really laborious, they demand extensive planning and big investments. If we apply this to animal research, we would impede every quick and dirty approach, every exploratory experiment to test something new - which research needs as well. |
|  |  | Rigid structure of ASR not satisfying the needs of research | | | W7 | W7 | RE3 But the only thing that I can think of that could be an issue with creativity is something I actually encountered in my own research, which is whenever you create a template that you have to fill in in order to do research, you in a way impose a structure on an activity that may not actually capture with adequate nuance the realm of different kinds of activities within that broader endeavor. |
| **Industry** | Intellectual property & competition | General issues | | | W8 | W8 | RE4 The weakness of this procedure is of course that preclinical research is very early in the development and that companies are really hesitant to reveal their data, or their secrets, at this point. You always feel that this is a big concern of the companies even in phase 1, they don't want their competitors to know how far they have got. |
|  |  | Re-analysis of study data from competitor | | | W9 | W9 | I2 If it happened that way, it would also mean that the companies could peek into each other’s data and could also spoil it. |
| **Overarching issues** | ASR not sufficient to solve reproducibility/translation issues | | | | W10 | W10 | I1 Regarding the publications of Amgen and Bayer, I understood them in a way that bias was only a small aspect but that the main aspect was that experiments weren't standardized enough. Principally, industry is obliged by GLP to accurately describe which reagent, which quality, how the experiment was run and so on. A very detailed description which is not found in academia. Admittedly, GLP is not compulsory at this early stage. |
| **Facilitators** | | | | | | | |
| **Preclinical/ Clinical Researchers** | Quality improvement by changing incentives | General issues | | | F1 | F1 | I4 And we should change our attitude, not reward research for the results that come out at the end but for the entire process. |
|  |  | Acknowledgement of ASR as a form of publication | | General issues | F2 | F2 | AR4 There is listed that I have 10 publications but not that I have 10 registry entries. I don't know how one could get a kind of positive rating for adding to this. |
|  |  |  |  | Current difficulties to "sell" negative data | F3 | F3 | AR8 We know from medicine that there is a trend to publish primarily positive results. One factor is that this is much more positively perceived by editorial boards and we know that the barriers for publishing positive results are much lower, this is well investigated. We have done this a few times and I can confirm that the barriers are much higher, the reviewers ask many more questions, why is it like this, have you considered that and so on. |
|  |  |  |  | Citability | F4 | F4 | AR4 It says I have 10 publications not I have 10 registry entries. I don't know how one could also count this as a kind of positive rating. [...] Not that negative, not like this didn't work and that didn't work, but more like a sort of publication that counts as well. |
|  |  | ASR as accreditation/ quality criteria | | General issues | F5 | F5 | AR1 the incentives is more like if you give them some accreditation, say like, okay you will get this accreditation, that’s mean you increase your level of expertise, you’ll go up one level. |
|  |  |  |  | Special relevance for particular countries | F6 | F6 | AR1 to say like we are in par with other countries like the West in animal work. Because that is one thing we say in Asia, because when we submitted our paper, they’re always, they’re worry that our result or our work is not up to the mark, they’re worried that we don’t use the standard practices |
|  | Intellectual property | Only quite general information registered | | | F7 | F7 | I4 The other thing is, I don’t have to, and then we’re getting again to the details of the animal registry, there’s no reason why all the details should be registered. So, when I list a clinical trial on clinicaltrials.gov, I do not relate the investigator brochure. So you can see that I’m doing a clinical study but you do not know how I am doing the clinical study. Only in the most general sense. So the same thing could happen here. I could list that I’m doing an animal study, and I am examining the EGF receptor versus the K-ras inhibitor, and I’m going to use a total of 100 animals and the experiment started on the first of January. And that would be all. And you wouldn’t know which EGF receptor inhibitor I’m using, you wouldn’t know which K-ras inhibitor I’m using, you wouldn’t know which strain of mice and so on. But the study would be registered. When it’s published, you would get a lot of that information but you still would never get the investigator brochure of the clinical study. You don’t have to reveal all of the information. It is a register to try to improve quality and reproducibility and animal welfare, not to check if you designed the study properly. That’s the job of your local ethics committee. |
|  |  | Post study registry | | | F8 | F8 | CR1 If there are results and they are not published because they are negative, not interesting, not suiting my concept or because the question changed, then the question is, when such data should be made public. Maybe one could define a project time, defined already in the proposal, saying for example that data should be entered one year after the end of the funding and be public as well. |
|  |  | Closed for appropriate time | | | F9 | F9a | I3 It will probably be better to define a cut-off date, for example if the experiment was performed 01.01.2015 then it should be available one or two years later. I would rather work with a time line. |
|  |  |  |  |  |  | F9b | CR2 I mean it is clear that everyone wants to get the information as fast as possible but you have to give those people time to evaluate and enter the data. |
|  |  | Inclusion of results after publication | | | F10 | F10 | CR1 For academic researchers it is important to have this protection until publication or abstract publication, that is an important good to me in the context of registries that we keep those data protected. |
|  | Resource considerations | Relatively small administration | | | F11 | F11a | CR1 I think when you have forms and provide those to the users and thereby ease the process, this could help a lot. |
|  |  |  |  |  |  | F11b | RE3 If you’re maintaining an electronic lab notebook now, the only difference, the only thing that would be time-consuming will be pressing the button that says “submit”. |
|  |  | Additional staff for administrative tasks | | | F12 | F12 | AR5 Incentives would be additional personnel for such administrative work. If one got an additional half or quarter position for someone just dealing with this. We are actually planning to use a researcher position for someone doing such administrative work, because we just can't cope with it anymore. |
|  |  | Financial reward for populating the registry | | | F13 | F13 | AR5 Well, sure, a form of financial incentive. |
|  | Needed information | General issues | | | F14 | F14 | RE3 And in those studies I would say that stipulating a sample size, stipulating your primary endpoint or something certainly around your primary hypothesis, the number of different arms of the study, the eligibility criteria for your animals, that would consist of what particular model you going to be using, what age of animals you are going to use, etc. Those to me, and some sort of contact information for the individual that is doing the research, those to me seem like, I guess off the top of my head, the minimal elements that you would want. Oh yes, and then of course, sorry, I didn’t mention, you want some information about the treatment, so you know whatever manipulation or intervention you are applying. By treatment I don’t mean a certain drug, I mean it could be a drug, but I just mean in any experiment you manipulate a system and by treatment I just mean a manipulation. Oh yes, and this is, I sort of said this but I just want to be more clear about it, your outcome measures, at least your primary outcome measure and preferably some of the secondary outcomes that will be measured. |
|  |  | Result registry | | General issues | F15 | F15 | AR2 And I think what is really necessary, are all results, also from the groups that are not published, not within the expected results. |
|  |  |  |  | Raw data | F16 | F16 | AR5 Raw data, exemplary, yes. Because then you can, when you look at a Western Blot or PCR, you can see how someone is working. If there is only a sloped, rudimentary band and N=2, then I wouldn't care for this. |
|  |  |  |  | Failures, negative results | F17 | F17 | AR8 Because it doesn't mean that a negative result is really negative. It may be for example that for Adenosine receptor A2 I find something completely different for the heart than for the liver. Originally my hypothesis was that stimulation of the receptor will have a similar effect to that in the heart. So at first sight this might be a negative result, but it might in a next step lead to my discovery of Adenosine receptor A3, which is responsible for the different effect on the liver. Therefore it doesn't mean because I cannot confirm my hypothesis that a result is negative and that there won't be a knowledge gain from this. |
|  | willingness in principle | | | | F18 | F18 | AR4 If I have done the experiment properly and it is just the substance showing no effect, then I have no problem in publishing it. |
|  | Peer and stakeholder pressure | General issues | | | F19 | F19 | I4 Well, I think it has to supported by the funding agencies or journals, otherwise they don’t have the … . So, it has to be done like it has with clinicaltrials.gov in the US that you can’t get a paper published in a top journal unless the study was pre-registered in …. And then journals like Nature, Science and Cell would have to refuse to publish a paper if it wasn’t pre-registered. And funding agencies would have to agree not to fund people who refuse to comply with an animal registry. So I think, you’ve got to get an agreement between the funding agencies and journals, that they are willing to make this compulsive. It’s not going to work if it’s just by choice, it has to be compulsive. |
|  |  | Question of familiarization | | | F20 | F20 | I2 In medicine we have to get used to more and more democracy and transparency and it is a process of customization. If one talked about study registries 15 years ago, people wouldn't get it |
|  |  | Current problems too obvious | | | F21 | F21 | I2 Because finally those drugs that are developed are used by the public. And I suppose that there will be a lot of resistance in industry, although some stakeholders might say that in principle you are right. |
|  |  | Attract interest of early career researchers | | | F22 | F22 | AR6 I am coping well with the existing system because I can judge it quite well. But when I was younger and new to the game, I could be very upset about people doing something and you later realize that this was not true, or that someone willingly backed the wrong horse, all those games. When you know the business, all that can't shock you anymore, you know it and just don't care about them. |
|  |  | Beneficiaries in research will lobby | | | F23 | F23 | AR2 You just have to have it started, so I think, once people start to write down the result and also the negative results, others will do it too and see that they benefit from it. I think it will grow, will start slow and if you’re lucky it will start to grow. |
|  | Protection of freedom of research | ASR for confirmatory, not exploratory studies | | | F24 | F24 | AR7 And I don’t think pre-registration would fit all types of experiments either. You know this whole thing that has recently been discussed about exploratory versus confirmatory experiments. And if you perform an experiment, you know, a confirmatory experiment, where you do power, sample size calculation, you do your inferential statistics and see whether a drug works or whether there is an association between a gene or whatever it is you are looking for, then I think registering those studies will probably more effective than all these exploratory experiments that people are doing. |
|  |  | Accessibility of ASR only for scientists/professionals | | | F25 | F25 | CR2 And that is a risk you have to assess clearly in such a highly differentiated field, so that you don't do more harm than good. Information: of course I want to know this, but it has to be interpreted by experts. That is a main point. I am clearly pro information, also in phase 1, I want to know as much as possible. But we should avoid the problem of a great amount of very specific data being offered to a broad community which is overburdened by this. |
|  |  | Anonymity ensured | | | F26 | F26 | AR2 Okay, the anonymous part is, mostly is the public, outside. Because you know, we have the researchers working on the animal experiments and we have people outside not understanding the animal research. They are really against it. And that’s always a big issue in doing the animal experiment actually. So it should be with a limited access, so not public but only for researchers, maybe research registry also and “anonymous” was in it. Maybe… For the positive result, I don’t think that it’s necessary but if it’s negative result, if somebody is publishing a mistake and everybody else saying “Oh my god how could he have done that?” So I’m very sure that nobody will write a mistake like that, honestly, in the registry if everybody else could see “this stupid guy did not think about, something everybody knows” to say it in an easy way. |
|  |  | Voluntariness | | | F27 | F27 | CR1 This would be a registry, basically, but voluntary, to help science, to show others what has been done on a voluntary basis. |
| **Research institutions** | Peer review/quality assurance of ASR | | | | F28 | F28 | AR5 And I think this is the difficulty, there should be someone to make sure that this registry, unlike PubMed for example, is crowded with data of no use to you because they don't reach the quality standards of your lab. And this would be another point, that someone would have to keep the registry clear, make sure that the data are useful to others, it makes sense to engage with it. |
|  | Institutions as controlling instances | | | | F29 | F29 | I4 So that meaning that you got to have some sort of check in place, that means the institutions will have to be involved in that because the journals can’t check and the funding agencies can’t check. But if I design the study, let’s say there are 4 animals in one cohort and 4 animals in another non registered with you, but I actually have already done the study. You won’t know that. It’s different from clinical studies which might run for several years. And you will know whether or not the study was pre-registered. That for an animal study would be really difficult. So you would also need the institutions involved, because they will have to check the laboratory notebooks to make sure that I pregistered the study and didn’t just register after I’ve already done it. |
| **Industry** | Attracts industry for identifying new ideas | | | | F30 | F30 | I2 It is not like the research labs of company X had made big progress in disease Y and the company had then made a medication from this. It comes from some researchers doing the work and the companies buy those results. And this process would be hastened by early transparency. |
|  | Moral benefit for drug industry | | | | F31 | F31 | I2 I believe that in the end it is a moral benefit for the companies, that industry will change and will be better, but that they sometimes lose money as well. But to sustain as an industry, they won't be able to avoid this. |
|  | Change in drug industry landscape | General issues | | | F32 | F32 | I2 It probably means as well the development of other industries, like it is already today that we are seeing specialists for phase 1 and 2 or preclinical research, who have their portfolio of products they sell, and the whole process of pharmaceutical development will be much more divided, which is starting already now. So there won't be one Pfizer, developing a product from the first idea, maybe collecting, let's say snake venom in Africa, to a marketable product 10 years later. Instead there will be companies doing only one step, e.g. selling molecule ideas or other developmental stages. Everyone will be much more under pressure to be fast, because the second they are start everyone else will know as well, therefore you can't play around for five years but you have to bring it to the market instantly. So there will be an acceleration and a lessening of monopolies. And that isn't bad from the population perspective. |
|  |  | Particular benefit for small companies | | | F33 | F33 | I2 Nevertheless I think that the "man on the street" or the small company, when they familiarize with the market, they have a better chance to assess things than if this weren't the case. |
| **Public/ Patients** | Public funders' interest in effective allocation | | | | F34 | F34 | RE1 for the… they who give the grant would important as well because they will never know what is been done what has already been done and in trials they now try to get the bigger picture and to know and to be up to date on what’s already been done but in those early trials there’s always of course dependent on what the researcher writes in his grant application: “this has never been done and I’m the first to do that, this will always work, it worked in cell cultures so it work in mice”, yes you say so, yes I think that would be very helpful those and for allocating the money to the right places |
|  | Public expectation of accountability in science | | | | F35 | F35 | RE1 I think the public would welcome a registry like that. Not so much in terms of I want to read it or I want to understand it but more in this feelings safe when things are transparent. |
| **IRB/ Regulatory Agencies** | Interest in unbiased data for reviewing phase I/II research | | | | F36 | F36 | RE4 We as an agency are more open, we would welcome it for our work if as much information as possible was provided in registries, because then you can really see: is this now the seventh study, the only one that went well and had a good safety profile, in contrast to the previous six studies that maybe didn't work out and could pose questions of interest to us. |
| **Journals** | Journals' interest in strengthened peer-review | | | | F37 | F37 | I4 And when I’m submitting to Nature, I can check all of those things, the study was performed blinded, the animals were randomized. But I do that after the study. You don’t know that I really did that. There is no way you’d know I really did that. I just can check the box and there is no way of knowing. The only way for you to know is if my university checks my lab notebooks. Because Nature is not going to do that. So you know, the very least, we got to have the journals, the funding agencies, the institutions all cooperating. I think this is probably a useful step going forward. |
| **Overarching issues** | Transparency as broadly shared intrinsic value | | | | F38 | F38 | I2 If this society does not completely degenerate, I am sure this will come one day. One cannot kill big ideas and the idea of transparency is a great one. |
|  | Consistency with existing governance tools | General issues | | | F39 | F39 | I1 We have information. Relatively late, I admit, from EPARs the European Assessment Reports. These describe the complete development of a medication. We have parts for fabrication of the active substance and of the drug, then a detailed part for preclinical data and a very detailed one for clinical data. Finally pharmacovigilance, adverse effects as well as risk-benefit-assessments. So we have all this information for drugs that were approved for the market. All the failed projects are missing here. |
|  |  | Combination with approval process for animal trials | | | F40 | F40 | I3 Everyone is using the same, simple system, which is even fused - now I come to something, making this easier than before - fused with the whole bureaucratic monster of animal protection, approval of animal trials. By combining this, I could reduce the work load. So I make one report which is used for animal protection and the registry, and my data are valuable both for animal protection and the registry, the approval process is accelerated and this all at least at a European level. |
|  |  | Knowledge from clinical trials registries | | General issues | F41 | F41 | AR1 But in the end I think, it could be really good for science at the end of the day. It definitely will be good for science. Because for the clinical trials, now we do have the standard procedure and protocol. The same way they have to do it for the animal work. |
|  |  |  |  | Acceptance of clinical registries | F42 | F42 | I1 I think that the discussions are almost finished. There are some rearguard battles, but I think we are on a good path. It is undisputed that we have registries now, regional ones for Europe, FDA and international ones and that all studies are registered and results should be published afterwards. |
|  |  | Ideas of Tox databases from industry | | | F43 | F43 | I1 I know considerations from big companies that say, couldn't we build a database, just for us, with access for the regulatory agencies that summarizes the tox data. But it is not concrete yet. However there was a two day symposium last year. I haven't followed up what happened to it. But it is an interesting initiative from the tox labs, the companies. It is open whether that will really happen, I think. But the idea was, opening the companies, at least for the involved companies and regulatory agencies, not going further for now. |
|  |  | Combination with "kill early and fast" policies | | | F44 | F44 | I1 In former times, molecules used to be taken into phase 3, even if you were lacking confidence in the substance and only then stopped. This has substantially changed. Phase 3 studies have become so expensive that companies are killing substances now already in phase 2 when they don't have a good feeling about it. That is why the failure rate in phase 2 has risen, partly dramatically, but fallen in phase 3. Economically and from patients' view this makes perfectly sense. |
|  |  | Combination with efforts for better reproducibility | | | F45 | F45 | I1 The better way of quality assurance in performing trials and also, what we as pharma companies are obliged to do, is validation. That is, a single result doesn't mean anything. Can be pure chance. We have to repeat our experiments at least three times, for clinical experiments twice to be able to show that this was not by chance but is a valid, reliable result. |
|  |  | Combination with more standardized basic research | | | F46 | F46 | I1 One could think about, although I am not sure how helpful this would be for basic research, applying the very strict rules of drug development to harmonize animal trials worldwide and improve data reliability. What I understood from these publications that would help, would be the better description of the experimental procedure, the reagents, the circumstances. |
|  | Alternatives/ Parallels to registries | Funders' journals | | | F47 | F47 | RE2 I think the type of thing that could work better I think are… a ‘Funders Journal’. This is done… there’s one funder in the UK that has its own journal and anyone that funded by this funder actually needs to publish the research in full in the journal. And then they can publish in other mentioned journal as well. They have to describe the whole study in the journal monograph… in the funder’s monograph. And that funder gets actually 98% of the research published and that is a drastic improvement from any other type of… any other research, really. (Interviewer interjecting ‘Yes’) And I think this actually could be something that improves publication bias… that is more effective than the registry. If all the funders had a journal, where the research is kept in full that can be a good start. That can be more manageable in the sense that it’s the same funder, it’s all in a one place. So the funders fund the research know exactly what is funded. They get the money for the progress. And they can have the result in house. I think that makes it feasible if it’s only in one place. (Interviewer interjecting ‘Yes’) I think that to me that would be actually more likely to have an impact. |
|  |  | Journal registries | | | F48 | F48 | RE2 there are different types of registry. One that I came across which actually would have an incentive is a registry similar to the registry that is held by the journals. So there is a format of research publication that is called registered report. When you register the protocol with the journal before you do the study. It is a kind of a registry but held with the journal that you’re registered with. |
|  |  | Journals for negative data | | | F49 | F49 | AR4 Or maybe that there are journals that also take negative data without a big fuss |
|  |  | Intensified coop: academia & industry | | | F50 | F50 | I1 Currently I see completely new cooperation between companies and academia. In former times it was more about contract research, or we just wait and then license a new biomarker or target. Now I see the foundation of shared laboratories. Or people from companies are sent to labs in which researchers from academia are working. The direct contact: What is important for industry? Where do the great ideas from academia come from? We want to combine these as early as possible, because can then make sure their quality criteria are met from the beginning. This is the future. |
|  |  | Efforts to better reproducibility | General issues | | F51 | F51 | I1 The most important thing we are building on is a very good basic research that is done with high accuracy and reproducibility. I know some scientific journals that want to found labs themselves to check previous to a publication or in parallel the reproducibility of the groundbreaking findings. That is one way. The down side in academia is that you won't win a Nobel Prize with a reproducing story. And a publication, yes, I confirmed the findings of professor XY, cannot be published in a high-impact journal. |
|  |  |  | Incentives for reproduction in academia | | F52 | F52 | I1 The most important thing we are building on is very good basic research that is done with high accuracy and reproducibility. I know some scientific journals that want to found labs themselves to check previous to a publication or in parallel the reproducibility of groundbreaking findings. That is one way. The down side in academia is that you won't win a Nobel Prize with a reproducing story. And a publication, yes, I confirmed the findings of professor XY, cannot be published in a high-impact journal. |
| **Barriers** | | | | | | | |
| **Animals** | Pressure from animal activists | General issues | | | B1 | B1a | RE3 So the first one is that animal research is notoriously controversial and transparency has a liability in terms of protecting research labs from harassment or pressure from various groups. So if you’re doing non-human primate studies or dog studies or, you know, cat studies, these tend to be the kinds of studies that generate the most amount of controversy. If you are required to register this in a public manner you are potentially advertising what is maybe controversial research and that may bring unwelcome attention to the research. |
|  |  |  |  |  |  | B1b | RE1 On the other hand, of course you will stir up the animal activist, and I’m not sure how that’s another thing I think preclinical researchers fear to have registry, I think that something which can’t be ignored. That you have a personal email, telling you “I will kill you because you kill mice” and know that those kind of email was sent to researchers. |
|  |  |  |  |  |  | B1c | RE1 I think so, because than you have of course, you have the numbers in the registry, of course you have to mention, there were 20 mice 3 dogs or 2 apes or whatever so people will come and use that data and will summarize and it’s not only just an assumption but then, this is really data. {Interviewer interject right, hard evidence so to speak} Right, and I think not only the known activist I mean there are known and heard and worked with to make things better, to void the animal experiment but those unknown private person that just now see “ oh my god, I didn’t know that” I don’t know.. |
|  |  | Better public understanding of animal research needed | | | B2 | B2a | AR2 I think in the US, it’s really known to the public that they’re doing animal research to increase the public health system. {Interviewer interjecting ‘right’}And even on TV, they have a kind of advertisement - pro animal experiment. And that is something that is missing in Europe or in Germany, for example. Yes so in here, more or less, you are doing the animal research behind the door and the public should not know about it. |
|  |  |  |  |  |  | B2b | AR7 Yes, I’ve heard that argument quite a few times and I just don’t really… especially now with this big push toward open access. And I think, in the late 80s, 90s there were especially in the UK, there were a lot of problems And we are much more open. And that suggests actually if you are open and transparent, if people understand what you are doing, then people… I think if you are secretive and you pretend you are not doing something and people find out, you will definitely get more backlash. No I don’t really see why if it was to happen why it shouldn’t just be open. |
|  |  |  |  |  |  | B2c | AR2 I must say that [research institution] is really good at publishing in regular newsletters about their success in therapies, in human therapies. They always said, “We did this for the first time and we had before animal experiments on it”. So that is really good at [research institution]. They’re really honest to the public that “We do an animal experiment and that humans benefit from it” |
|  |  | Cultural differences | | | B3 | B3 | AR2 You know, in Germany or in Europe everybody has a good living standard and in countries where people are poor and have other problems like wars and there, the animals welfare isn’t an issue. |
|  | Higher sample sizes as unintended consequence | | | | B4 | B4 | CR1 I see a possible danger in having to perform very large experiments then because you have to register and say I am doing this definitive experiment, overlooking that you are using the wrong mouse strain - which you would have noticed in a pilot study. Meaning that maybe you do valid studies with large groups but you also use more animals. And more effort because maybe I cannot perform pilot experiments any longer when I have to register every experiment beforehand. |
| **Preclinical/ clinical research** | Resource considerations | Fear of more standardization/regulation | | | B5 | B5 | CR1 As a researcher I am a bit afraid of such a new regulation demanding that I register every study in advance, doing statistical calculations, maybe even with a statistician, then performing the study and afterwards I have to report everything. |
|  |  | Lack of time to use registry | | | B6 | B6 | AR5 Yes, but have you worked yourself through those big groups with all their models? This is the work of a lifetime. I think this is unrealistic. To have a benefit, taking you forward in your work, you have to build small groups, you know the people with whom you can work together, you can discuss what is working and what not. And then you know how to evaluate the results. |
|  |  | Fear of additional administration | | | B7 | B7 | RE2 I think that if the registry involves too much bureaucracy you are going to lose a lot of people along the way. |
|  | Unclear benefit from a registry | | | | B8 | B8 | RE 2 I think another disincentive is that, it have never been… I have never seen clinical trial preregistration presented as a real means to reduce the publication bias. But at the end of the day, we know that it helps quantify it but it doesn’t really reduce it. If you look at the clinical field, it is not… there is no clear evidence that it has reduced the publication bias and it’s not the real aim for it - the aim is actually trying to quantify the problems rather than reducing it. So I think for preclinical researchers, that also work as a disincentive because they are not quite sure that this is asking them to do more work on top of what they’re already do. And the result is not really clear. And I think if there was a clear explanation on how it would actually reduce the problem, I think it might be a great incentive for researchers. But most preclinical researchers don’t really get either systematic reviews or that kind of thing anyway. So they’re not going to… if you tell them that it really important to have the systematic reviews can include studies that are not published. They don’t… most of them don’t do systematical reviews, that is such a high walk. That person doesn’t really talk to them. So I think that should be a problem. |
|  | Limitations to publish in journals | General issues | | | B9 | B9 | RE1 if you can’t publish a paper if u first published it in the registry. And we discussed this with [....] and the publisher or some of the publisher sort of said, “Yeah, if just those 20 items are published, we won’t regard it as a publication”. But this is of course an issue if you can’t have a paper because you published your result earlier. That’s of course a big barrier. |
|  |  | ASR as pretext for paper rejections | | | B10 | B10 | RE1 If they’re not interested in the results because these are negative result or they think this is a boring thing then they come up with registry “well, you haven’t registered it in advance, I’m sorry but I can’t publish your paper” |
|  | Difficulties to define start/end of experiments | | | | B11 | B11 | I3 And then you have the question to what project does an experiment belong? One can be creative in this, there is always more than one wyy to assign an experiment to a project. There is the main project, the follow-up project, side projects - and at will one could jump from one to another. Having this in mind, the most fair and reasonable method would be a deadline, I think one or two years should be no problem. |
|  | Difficulties to define study subgroups requiring registration | | | | B12 | B12 | AR9 Unfortunately, in preclinical research people don't make the distinction between exploratory and confirmatory. Many are working exploratorily and combine it with confirmatory but they don't determine what it is. |
|  | Unclear trust in ASR funder | | | | B13 | B13 | AR9 Trust and what is behind it, who is doing it. That is a question always coming up, who will be the one really operating such a registry. For clinical studies it is more obvious whose responsibility it is to do this, NIH or whoever, but in the end it is an organ of the American government. |
|  | Lack of "failure culture" | General issues | | | B14 | B14a | RE2 The issue that research that is impactful is valued over research that is robust and of quality. So that is a major problem. That the researchers are looking for the impact than the quality. And impact is valued over the quality. I think that is a massive issue. Because at the end of the day, the negative studies is as valuable as the positive studies. |
|  |  |  |  |  |  | B14b | AR2 For the positive result, I don’t think that it’s necessary but if it’s negative result, if somebody is publishing a mistake and everybody else saying “Oh my god how could he have done that?” So I’m very sure that nobody will write a mistake like that, honestly, in the registry if everybody else could see “this stupid guy did not think about, something everybody knows” to say it in an easy way |
|  |  | Fear of funding/career disadvantages | | | B15 | B15a | AR4 In principle, I like the idea but I think that scientists would struggle with entering their negative data there. And maybe, if someone had more negative data and had his name written there, then it is like, oh, for example the DFG looks in there and says, oh, he has a lot of negative data. I don't know if we should fund him. |
|  |  |  |  |  |  | B15b | CR1 When you apply for a job and say I always backed the wrong horse, then this is unpleasant of course. |
|  |  | Reputation | | | B16 | B16 | AR6 The majority is classically written down and I would say it is hard to communicate this to others, if you upload them now or show it years later to others, because it also shows the meandering of such projects which often don't just go straight from beginning to end. And I would be concerned to show this all to the public. |
|  |  | Reluctance to submit failed studies | | | B17 | B17 | AR2 I would be afraid that not everybody is really honest in writing down their errors or failures. And so it should be anonymous in some kind, I don’t know how far anonymous it can be because we’re talking about research data and there’s always a competition between groups. I think that is a big obstacle, to get honest information from researchers |
|  | Aversion regarding change in general | | | | B18 | B18 | I4 The biggest hurdle will be investigators’ reluctance. So, now I was involved in many clinical trials in the 1970s and in the 1970s the way clinical trials were conducted is exactly the way we do preclinical research at the moment. There is no control, I can pick and choose the patients, in the 1970s, I can exclude them if I want to, I can change the protocol. In fact, in the 1970s many people were included in clinical trials without even knowing that were on clinical trials. That is a horror today. But that is like it was 50 years ago. So, that’s what it’s like today in preclinical area. The second biggest hurdle was getting physicians to accept the changes. So there many physicians, who said, clinical trials will be, creativity will stop, research will cease if we got to get patient consent, if we got to get approval from the institutional ethics committee, if we got to randomize patient. You know I’ve been at many meetings with people that said, we cannot do this study if we have to randomize patients. That’s not the case today, but 30 years ago I was in many, many teams where physicians would say, we can’t randomize patients in this study, if we do this study will make a go of it. ….. has changed. But the biggest hurdle was getting physicians to accommodate to that change. |
|  | Ignorance of general interests of scientific community | | | | B19 | B19 | RE1 I find it very, you saw very often that people thought “Ok, we have this hypothesis; We’ve thought this might have worked but it didn’t worked so nobody will be interested in the result.” So this one more or less self-censorship, not even trying to get the result out because you think it’s not interesting because it’s not working. This might be a new vaccines, this might be a new whatever you are just looking at in physiology or cancer physiology of what we are doing, so I think, the first thing is in your head, you have to filter and think nobody is interested in that, it didn’t work, my idea didn’t work out. Not thinking that this is of course as well interesting for the others because I might have to say the idea which actually is not that rare, but people have the same idea what may worked or may not worked in preclinical work. |
|  | Ignorance of non-scientific interests | | | | B20 | B20 | RE1 We’ve thought this might have worked but it didn’t work so nobody will be interested in the result. |
| **Research institutions** | Lack of institutional support | | | | B21 | B21 | RE1 I think those people I still have contact with right now and meet with at the conferences and behind the closed door, “we don’t have any problem with it”. But on the public they can’t say be this industry or be in the academia. Because they would get trouble with their bosses but the people really on the bench, they have no problem with it. |
| **Industry** | Reluctance to take up benefits of registry | | | | B22 | B22 | I2 I'm always stunned when I visit companies, telling me about the competing product, not knowing that all the numbers for the whole country per department are available publicly. But that is the fate of transparency, that only a minority really dives into the data and makes use of them. |
|  | Potential lobby against ASR | | | | B23 | B23 | I2 A thousand lobbyists will list these arguments, why it won't work, and it is clear that the industry behind has enough money to pay people for going onto a stage and telling stories. |
|  | Intellectual property | | | | B24 | B24 | CR2 Industry is focused on earning money with these ideas and to implement it accordingly. Here, different views are colliding. If industry sponsors animal trials and the experiments don't show the desired results, they are going to keep it and won't share it with the scientific community, also to protect patents. These considerations, especially the view on patents and the extremely complex processes involved - you first have to apply for a patent, this is examined, some days it is published, and one to two years are gone. During this time people that want to earn money have no interest in sharing any information. |
|  | Competition | | | | B25 | B25 | I2 As an economist one would say, everyone needs a little monopoly power. When the market is completely transparent no one is going to make profit. |
|  | No opt-out option | | | | B26 | B26 | I1 When there is a registry, it is clear that this must apply to all preclinical research. There shouldn't be a registry for academia and industry is out. That is neither sensible nor politically feasible. |
| **Regulators/ IRBs** | Hesitant in approving same/similar research | | | | B27 | B27 | AR5 I mean if that later impedes you, because you don't get approval for your animal trial application, this would give you the whole nine yards. This should be excluded. If you want to do a redundant experiment, then you should have the right to do it without arguing with anybody. Because researchers need some freedom of research. |
|  | Lack of time to use registries in reviewing clinical trials | | | | B28 | B28 | RE1 We assume that those ethics committees look at those registries before they discuss about a submitted new clinical trial but that’s not the case. That would be of course very helpful, it was one of the reasons we did that, so in the same of course true with those studies, I mean if you do your work well and have time and resources for it, if you have to really look at an application, you should know what has been done already, and again not what’s the researcher said but which you can verify |
| **Overarching issues** | Resource considerations | Costs | | | B29 | B29 | RE1 Academia are far worse in that perspective because they don’t have the infrastructure, they don’t have the resources, it’s an add-on task that they have to do |
|  | Possible limitations of the value of a registry | Searchability of a registry, amount of data | | | B30 | B30 | I3 In the worst case it is a huge database that no one uses and that helps nobody because no one can handle it, you can’t find anything or you need to be extremely trained to use it. |
|  |  | Plurality of ASRs | | | B31 | B31 | I1 We wouldn’t like parallel databases with the same content. That means a great deal of work for the companies. We see this for the clinical trials: you have the FDA database, the European database and national databases in Germany, France, Spain. This means a huge workload. The companies pay people for redundant work that is in the end pointless. |
|  |  | Technical issues, compatibility of formats | | | B32 | B32 | I3 The second point is, I am quite sure that the documentation standards and technicalities are quite different between companies and even more in academia. So if we have 10,000 formats in 5,000 programs this is not workable. Therefore we need a kind of revolution to standardize it, to say, every animal researcher in the world have a form of lab notebook and we now agree upon a certain standard and everyone uses the same software, enters the same values and then we can create something that has value. |
|  |  | Value of ASR only comes with up-to date information | | | B33 | B33 | AR6 One would have a look in the beginning and then the question would be, how up-to-date is it, how good and then it would be used. It can have a certain value, if I know I can look things up there, it is up-to-date, I get useful and good information there, then people will make use of it. If I see that people only put very old data in, that is something different. |
|  |  | Value of ASR only comes with widespread, representative population | | | B34 | B34 | AR9 It has to be a full representation of what is going on, otherwise we don't need it, it makes no real sense. It only works, I believe, and can only achieve the good I mentioned and help to fight those nuisances, if it has a strong, if not essentially perfect, coverage of the preclinical field. |
|  |  | Value of ASR only comes with detailed information | | General issues | B35 | B35 | RE2 So, you know… maybe we could do better with the animal research because we actually have these minimum information required from the very start. But I think at the minute, there’s an issue with the type of information that’s presented. There are lots of entries that don’t have the minimum information that you need to follow up. |
|  |  |  |  | Raw data | B36 | B36 | I2 In an ideal world, one could really prove study results, by raw data in some form, not only that the IQWIG can show you data upon request, but that it is in some form available to the public. So that basically everyone could recalculate, because study bias, I am convinced, is not only produced by studies that are not published at all, but also by the huge opportunity to manipulate data analysis, that only becomes visible if you really analyze the data yourself. Then you can see what strategies were used in the publication to leave out or shading data, let alone fraud - if you don't see leaving out and shading data as fraud. |
|  |  |  |  | Problems with low data quality/ inconclusive data | B37 | B37 | RE3 Registries are highly variable in terms of their quality, so there are numerous registration records in clinical trial registries that are problematic and un-informative. And registries are really only as good as the level of curation in the registries, or the level, extent to which you can trust the content in them. |
|  | New source of bias | Potential bias by variation of registration practice | | | B38 | B38 | RE2 I think even with the high impact factor for clinical trial, for some journal is like PLOS are started to accept the registration, which is then after the study. So they’re really… they want basically a registration number. Then afterward, they will still accept the paper. And I think they actually have to compromise because they implemented it really strictly. |
|  |  | Potential new bias by rejection of non-registered studies | | | B39 | B39 | RE2 there’s no evidence that studies that are not registered have a low quality, we don’t have that evidence yet. And there’s a risk that you have created another type of bias by being really strict in accepting the one that has already been registered. So I think it would be a problem even if it were mandated, it’s not going to that simple. |
|  | Unexpected user groups, scientific lay people | | | | B40 | B40 | CR2 The point of transparency is simple: we have similar problems in medicine: the more data is published, the more lay people will use it. I have talked about venture capitalists before. Is it good that every banker, every economist starts looking into the data before financing an early clinical trial? Are they really skilled to assess that information? Or shouldn't we leave that to expert who have the knowledge to evaluate those data? |
|  | Difficult change management | | | | B41 | B41 | AR1 You’re saying it is difficult. I see that what is happening in the institutional around us, when there is a change, the initial first thing people think is “no no no - it is not going to work”, so getting them to accept it and you know, they’re making it work is the most challenging part. |
