# Supplementary material for: Animal Study Registries: Results from a Stakeholder Analysis on Potential Strengths, Weaknesses, Facilitators, and Barriers
Source: PLoS Biol. 2016 Nov 10;14(11):e2000391. doi: 10.1371/journal.pbio.2000391 (PMC5104355; doi:10.1371/journal.pbio.2000391)
Supplement: S2 Table — (DOCX) [file pbio.2000391.s002.docx]

**A****nimal study registries. Results from key informant interviews on potential strengths, weaknesses, facilitators and barriers**

Susanne Wieschowski^1^,PhD, Diego S. Silva^1,2^, PhD, Daniel Strech^1^, M.D., PhD

1 Institute for Ethics, History and Philosophy of Medicine, Hannover Medical School, Carl-Neuberg-Strasse 1, 30625 Hannover, Germany

2 Faculty of Health Sciences, Simon Fraser University, Blusson Hall, Room 11008, 8888 University Drive, Burnaby, B.C., Canada, V5A 1S6

Corresponding author:

Daniel Strech, Institute for Ethics, History and Philosophy of Medicine, Hannover Medical School, Carl-Neuberg-Strasse 1, 30625 Hannover, Germany

Phone: +49 511 532-2709/-6498

E-Mail: strech.daniel@mh-hannover.de

| **Paper**  Table S2  **Table S2** | **Statement that Animal Study Registry (ASR) should exist** | **Ethics Argument in Favour of ASR** | **Questions and comments on process for ASR** |
| --- | --- | --- | --- |
|  |  |  |  |
| Roberts I, Kwan I, Evans P et al. (2002) Does animal experimentation inform human healthcare? Observations from a systematic review of international animal experiments on fluid resuscitation.  *BMJ* 324: 474-476. | “Prospective registration of animal experiments would help to avoid publication bias” |  |  |
| Perel P, Roberts I, Sena E, et al. (2007) Comparison of treatment effects between animal experiments and clinical trials: systematic review.  *BMJ* 334:197-203. | “Prospective registration of animal experiments might reduce publication bias” |  |  |
| van der Worp H, Howells D, Sena E, et al. (2010) Can animal models of disease reliably inform human studies? *PLoS Medicine* 7(3): e1000245. | “… we suggest that [existing animal regulatory frameworks] might be exploited to allow the maintenance of a central register of experiments performed” |  |  |
| Korevarr D, Hooft L, ter Riet G. (2011) Systematic reviews and meta-analyses of preclinical studies: publication bias in laboratory animal experiments. *Lab Anim* 45: 225-230. | Rise of meta-analysis and systematic reviews of animal studies may lead to a rise in the call for ASR as has been the case in the clinical research realm. |  |  |
| Ioannidis J. (2012) Extrapolating from animals to humans. *Science Trans Med* 4(151): 1-3. | “Consider preregistration of animal studies (especially experimental trials)” |  |  |
| ter Riet G, Korevaar D, Leenaars M, et al. (2012) Publication bias in laboratory animal research: a survey on magnitude, drivers, consequences and potential solutions. *PLoS One* 7(9): e43404. | “A system ensuring periodic follow-up of each experiment’s fate would reinforce such [animal studies] registration” |  |  |
| Hooijmans C, Ritskes-Hoitinga M. (2013) Progress in using systematic reviews of animal studies to improve translational research. *PLoS Medicine* 10(7): e1001482. | “An international initiative to register animal studies must be started” |  |  |
| Begley G, Ioannidis J. (2015) Reproducibiilty in science: improving the standard for basic and preclinical research. *Circ Res* 116:116-126. | “The notion of increased oversight of preclinical studies has long been proposed” |  |  |
|  |  |  |  |
| Kimmelman J, Anderson J. (2012) Should preclinical studies be registered? *Nature Biotech* 30(6): 488-489. |  | "Helps secure welfare of individuals and institutions that rely on access to evidence" |  |
|  |  |  |  |
| Varga O, Hansen A, Sandoe P, et al. (2010) Improving transparency and ethical accountability in animal studies. *EBMO Reports* 11(7): 500-503. |  |  | Pros: standardize information gathered; help combat publication bias  Cons: deciding on relevant info to include; cost; violence against animal researchers because of publicity |
| Jansen of Lorkeers S, Doevendans P, Chamuleau S. (2014) All preclincal trials should be registered in advance in an online registry. *Eur J Clin Invest* 44: 891-892. |  |  | Registry should include: basics of study design; secure and limited access to researchers and registry staff until completion of study; allow for disclosure of results; journals ought to demand it. |
