# Supplementary material for: Animal Study Registries: Results from a Stakeholder Analysis on Potential Strengths, Weaknesses, Facilitators, and Barriers
Source: PLoS Biol. 2016 Nov 10;14(11):e2000391. doi: 10.1371/journal.pbio.2000391 (PMC5104355; doi:10.1371/journal.pbio.2000391)
Supplement: S1 Text — (DOCX) [file pbio.2000391.s003.docx]

**Interview Guide – Animal Study Registration**

NB: The purpose of this guide is to do just that – guide the interviewer. The goal is not to be exhaustive necessarily but to make sure that key information is gathered with respect to the goals of the project and the individual you’re interviewing. For the more specific questions, therefore, do not read out the guide verbatim; rather, the guide is meant only to remind you what you need to ask during the interview. Try to be casual and adopt a conversational style with your interviewee.

For the introduction of the project, however, stay close to the information and balanced wording described in this guide. It is important that the project is introduced in similar way to all interview partners.

[Introduction – General Remarks, prior to turning on the recorder:]

[Introduce yourself and thank participant for being part of the project ]

The funder for this project is the German Research Foundation

The Principal Investigator is Daniel Strech, and the research team consists of members trained in ethics, regulatory sciences, and bench science

We will be asking you a series of questions; please answer as freely and honestly as you can

The interview will take about 30-45 minutes and will be audio recorded so that we can analyze the text later

Do you have any questions?

[Make sure you then get them to sign the consent form]

[Turn on the recorder]

[Introduction – Remarks about the project]

[Speak slowly, use conversational but descriptive and exact language]

Increasingly, there exists evidence to suggest a substantial problem with publication bias leading to a lack of reproducibility in preclinical research, including animal studies.

Publication bias can occur in different ways:

For example, results from completed studies or projects are not getting reported (for several more or less legitimate reasons), particularly negative or null results.

Another example is that published studies preferably report positive results, or selectively report positive and significant findings.

The problem of publication bias and reproducibility in preclinical research can have several implications. For example, some argue that the relatively high frequency of failures in phase I or II clinical trials can, at least to some extent, be traced back to publication bias in preclinical animal research. The high rate of failure of phase I and II in turn provokes the question whether publication bias in animal research undermines the adequate protection of human participants in the early stages of clinical trials. Last, but not least, publication bias might lead to the waste of animals in preclinical studies due to redundant studies being conducted stemming from unreported results.

[PAUSE, then]

So, most people who think about how to reduce publication bias believe that it will require several intertwined approaches and that different stakeholders need to take on different roles in making this possible. From all these potential approaches, our study focuses on the issue of ANIMAL STUDY REGISTRIES [emphasize and speak slowly]. In the last 2 years several scientists, as well as those who work in regulatory matters, brought up the need of animal study registries.

What do we mean by such animal study registries? It would be relatively similar to the well-established registries for clinical trials such as clinicaltrials.gov in the US or the WHO registry for clinical trials. A registry for animal studies would allow, OR even require, animal researchers to register some project-specific data in a certain database. What study specific data should be registered and when, along with the time at which these data will be made public still is an open question.

The registered studies would in turn allow other animal researchers to check whether their own idea for an animal study has already been investigated and whether it would be a waste of resources to conduct an experiment. OR clinical researchers who are motivated to design a phase I or II study because of positive findings from published animal studies could check in such animal registries whether or not other finalized studies to the same question exists that were not reported in the literature. ALSO funders, regulatory agencies or journal editors could be interested to check such registries for several purposes.

[PAUSE, then:] We are conducting this interview with you and we are conducting further interviews with different stakeholder to better understand what are the POTENTIAL [emphasize] strengths and weakness of animal studies and what are potential barriers and facilitators of their effective implementation.

General questions; meant to give interviewee space to get comfortable being interviewed. Be attentive to probing or potential follow up questions given what he or she says.

- What do you think might be some of the strengths and weakness of a registry for animal studies?
- What type of things would you want to see in an animal study registry? What do you think is important or are “must-haves”? Why do you think this is the case? Can you give an example?
  - Further probing question: specifically, is there anything you think should not be in a registry?
- How important do you think the registration of animal studies will be for improving the transition from preclinical to clinical trials, in terms of publication bias and issues around reproducibility?
  - *Further probing question*: why do you think this is the case? Can you give an example?
- Practically speaking, what are potential barriers or facilitators for the implementation of animal study registries?
  - *Further probing question*: You mentioned some barriers (or facilitators) do you have some facilitators (or barriers) in mind?
  - *Further probing question*: You mentioned xyz. Do you have any suggestion how to overcome this problem?

Specific questions –questions that should be answered, but can arise through the interviewees’ answers to the previous set of questions. These questions can be presented in any order to the interviewee:

ANIMAL RESEARCHERS

- Some animal researchers argued that study registries would negatively affect creativity in preclinical research. Others opposed to this issue. What do you think?
  - Can you give an example?
- What would need to be in place for you to agree to use a studies registry?
  - What incentives should be used to get animal researchers to use registries?
  - To what extent do you think the culture surrounding animal research must change, for example, the ‘publish or perish’ mentality?

CLINICAL RESEARCHERS

- To what extent do you feel that an animal trials registry would improve preclinical trials evidence?
- To what extent do you feel that an animal studies registry would improve planning and design of clinical studies?
- Have you used information from clinical trials registries to improve study design? Why or why not?

INDUSTRY

- What aspects would have to be in place to make an animal studies registry worthwhile?
- Would there be any intellectual property issues that ought to be considered?
- Do you think that registries help to minimize the failure rate of clinical trials and costs in drug development? What would be their impact?
- From an industry perspective, would you favor the registries for university based registries or would you favor other approaches to minimize publication bias?

Final Question:

- Anything else you think we need to consider moving forward? Anything we missed?
