# Supplementary material for: Animal Study Registries: Results from a Stakeholder Analysis on Potential Strengths, Weaknesses, Facilitators, and Barriers
Source: PLoS Biol. 2016 Nov 10;14(11):e2000391. doi: 10.1371/journal.pbio.2000391 (PMC5104355; doi:10.1371/journal.pbio.2000391)
Supplement: S2 Text — (DOCX) [file pbio.2000391.s004.docx]

Dear …,

We are a research group from Hannover Medical School (Germany) that investigates issues in research governance (www.mh-hannover.de/cells-strech-group.html?&L=1). In a current research project funded by the DFG (German Research Foundation) we address the issue of publication bias in animal research. More specifically, we investigate how the implementation of registries for animal studies could help to minimize publication bias and how it could be beneficial or detrimental to the scientific community and public. This investigation includes conducting interviews with key informants who could help elucidate ideas and challenges related to registries for animal studies. The primary objective of this interview study is to assess the full spectrum of potential strengths and weaknesses of such registries as well as factors that could facilitate or inhibit an appropriate registry implementation procedure.

You have been identified as someone who could meaningfully participate in interviews on this topic. Therefore, we would like to invite you to participate in a 30-45 minute interview. The benefit of your participation is to contribute information that supports debate and policy decision making with regard to registries for animal studies. There is a €100 honorarium associated with participation.

Background

Increasingly, there exists evidence to suggest a substantial problem with publication bias leading to issues related to a lack of reproducibility in preclinical research, including animal studies. This problem leads to several economic and ethical challenges that are currently discussed by animal and clinical researchers, research institutions, public and private funders, regulators, bioethicists and policy decision makers. Addressing the problem of publication bias in preclinical research will likely require the interplay of several established and new approaches. Registries for animal studies were proposed by several experts to be one of these new approaches; however, other experts had strong reservations regarding the use of such registries.

Next steps

Please let us know whether you agree to participate in this project. Your participation is fully voluntary. All your personal data (i.e., name and affiliation) will be de-identified and kept in strict confidence. We will disseminate findings from this interview study in a way that will not allow you to be identified neither as the interview participant nor your affiliation. We are happy to work around your schedule and to meet you wherever is best for you. Finally, please find enclosed in this email a copy of the informed consent form.

Best wishes,

Daniel Strech MD, PhD (Professor), Susanne Wieschowski, PhD (Research Fellow), Diego Silva, PhD (Research Associate)
