# Supplementary material for: Animal Study Registries: Results from a Stakeholder Analysis on Potential Strengths, Weaknesses, Facilitators, and Barriers
Source: PLoS Biol. 2016 Nov 10;14(11):e2000391. doi: 10.1371/journal.pbio.2000391 (PMC5104355; doi:10.1371/journal.pbio.2000391)
Supplement: S3 Text — (DOCX) [file pbio.2000391.s005.docx]

**Consent Form**

**Information and Purpose**: The purpose of the study being conducted is to investigate how the implementation of registries for animal studies could help to minimize publication bias and how it could be beneficial or detrimental to the scientific community and public. This investigation includes conducting interviews with key informants who could help elucidate ideas and challenges related to registries for animal studies. The primary objective of this interview study is to assess the full spectrum of potential strengths and weaknesses of such registries as well as factors that could facilitate or inhibit an appropriate registry implementation procedure.

**Benefit and Risks**: The benefit of your participation is to contribute information that support academic debate and policy decision making with regard to registries for animal studies. A summary of the results will be available to participants upon request. There are no risks associated with participating in the study.

**Your Participation**: Participation consists of one interview, lasting approximately 30-45 minutes. This interview will be audio taped. There is a €100 honorarium associated with participation.

Your participation is fully voluntary and you may withdraw participation at any time without penalty.

**Confidentiality**: All your personal data (name and affiliation) will be de-identified and kept in strict confidence. We will disseminate findings from this interview study in a way that will not allow you to be identified neither as the interview participant nor your affiliation.

**Contact:** Please contact Dr. Daniel Strech by e-mail at strech.daniel@mh-hannnover.de or by telephone at +49 511-532-4278 with any questions or concerns.

___________________________ ______________________________

Printed name of Interviewee Signature of Interviewee

___________________________ ______________________________

Printed Name of Interviewer Signature of Interviewer
